# Comparison of the effects of Diplen LX membrane and Coe-Pak on pain, wound healing, and patient preference after the periodontal flap surgery in patients with moderate to severe chronic periodontitis

**DOI:** 10.34172/joddd.2022.023

**Published:** 2022-10-15

**Authors:** Mehrnoosh Sadighi, Masoumeh Faramarzi, Reza Pourabbas, Zeinab Torab, Hamidreza Mohammadi, Salar Hoseein Nazmi

**Affiliations:** ^1^Department of Periodontics, Faculty of Dentistry, Tabriz University of Medical Sciences, Tabriz, Iran; ^2^Department of Pediatric Dentistry, Faculty of Dentistry, Tabriz University of Medical Sciences, Tabriz, Iran; ^3^Private Practitioner, Urmia, Iran

**Keywords:** Coe-Pak dressing, Diplex LX membrane dressing, Pain, Periodontal flap, Periodontitis, Wound healing

## Abstract

**Background.** After periodontal surgery, in most cases, the surgical area is covered with a surgical pack. It has been suggested that these packs might minimize complications. This study aimed to compare the effects of Diplen LX membrane and Coe-Pak on pain, wound healing, and patient preference after a periodontal flap surgery in patients with moderate to severe chronic periodontitis.

**Methods.** In this randomized clinical trial, 26 patients were evaluated. Pain scores were assessed using visual analog scale (VAS) on the 3rd and 7th days postoperatively and compared between the two dressings. On the 7th and 14th days after both flap surgeries, surgical site healing was evaluated using the wound healing index (WHI).

**Results.** The mean age of the patients was 31. It was observed that the severity of pain in the studied patients on the 3rd and 7th days postoperatively was significantly lower in the intervention group than in the control group. It was also observed that the value of WHI in the studied patients on the 7th and 14th days postoperatively was significantly higher in the intervention group than in the control group.

**Conclusion.** The pain was less severe in both groups using periodontal dressing and also lower in the Diplen LX membrane group. In addition, based on WHI, wound healing score in patients was also higher and more favorable in the Diplex LX membrane group. Due to the above factors, the majority of patients preferred the use of the Diplen LX membrane.

## Introduction

 After periodontal surgery, the surgical site is often covered with a surgical pack. It has been suggested that packs might minimize surgical complications, such as postoperative infection and bleeding, facilitate tissue healing by preventing physical traumas during chewing and speaking, and prevent granulation tissue formation.^1^ Periodontal dressing was first introduced in 1923 by Ward. Wonder Pack was a substance based on zinc oxide eugenol used to cover and protect the surgical site.^1^ Some researchers have examined periodontal dressings. The purpose of periodontal dressings is to control bleeding and discomfort after surgery, splint loose teeth, provide aseptic conditions for tissue healing, prevent pocket re-formation, and desensitize cementum.^2^ In non-surgical procedures, the use of periodontal dressing can be helpful in aggressive periodontitis patients.^3^ A review of the literature shows that periodontal dressing makes the patient more comfortable after surgery and reduces the dead space of the periodontal flap. Therefore, it is used to cover and protect the wound from the external environment, protect the denuded bone during the healing process, and splint mobile teeth after surgery.^4^ Coe-Pak is one of the most common periodontal dressings and is used as a standard for comparison with other dressings. Coe-Pak is a two-component dressing without eugenol, which also contains bacteriostatic agents. In addition to the common properties of all periodontal dressings, it lacks tissue stimulants, such as eugenol, and has good adhesion properties,^5^ adheres well to teeth and soft tissues, and prevents the flap from detaching from the root surface.^6^ Despite its widespread use, its disadvantages include poor appearance, indeterminate setting time, and poor flow characteristics during preparation. In addition, its bulky and delicate appearance has always been a problem.^7^ Diplen LX is a new absorbent membrane with two absorbable and non-absorbable layers. Its hydrophobic layer has texture-compatible properties and contains hydroxyapatite and calcium phosphate. Its hydrophilic layer adheres firmly to the bone surface in the area of bone defects and remains for weeks. Its optimal adhesion properties allow it to be used without the need to use other adhesive elements.^8,9^

 Primary periodontal dressings used to cover and protect the surgical site immobilized wound areas, controlled bleeding, created aseptic conditions for tissue repair, and physically protected the wound and its contents, leading to better repair. Coe-Pak is one of the most common periodontal dressings, which despite its good properties, has disadvantages such as poor appearance, indeterminate setting time, and poor flow characteristics during preparation. In comparison, Diplen LX, in addition to having common properties of periodontal dressings, is a type of biopolymer that can be absorbed as a transparent adhesive film and does not have the disadvantages of previous dressings. Therefore, this study aimed to compare the application of Diplen LX and Coe-Pak membranes in terms of patient preference, pain, and wound healing after a periodontal flap surgery in patients with moderate and severe periodontitis.

## Methods

 This split-mouth, single-blind, comparative study involved patients with chronic generalized periodontitis. All the rights of human subjects were observed in this study. After the approval of the Ethics Committee of Tabriz University of Medical Sciences, the procedures were undertaken. This study was approved by the Ethics Committee of Tabriz University of Medical Sciences under the code IR.TBZMED.REC.1398.707. Thirty adult patients of either sex aged 19–54 years, with generalized pocket probing depths (PD) of ≥5 mm, requiring periodontal flap surgery in at least two different quadrants, were selected randomly for the study. Written informed consent was obtained from each subject after explaining the proposed study design, treatment outcomes, potential risks, and benefits.

 Patients with systemic diseases such as tuberculosis, uncontrolled diabetes mellitus, hypertension, etc., which could influence the outcome of the study, pregnant and lactating mothers or those planning pregnancy and smokers were not included in the study. A detailed history was taken, and examinations were carried out along with a complete hemogram and panoramic radiographs. Thirty patients were subjected to phase 1 periodontal treatment that included thorough supragingival and subgingival scaling and root planing. The patients were placed on a strict oral hygiene maintenance program. Re-evaluation was carried out 4–6 weeks after completing phase I therapy, and baseline clinical parameters, i.e., plaque index (PI),^10^modified sulcular bleeding index (mSBI),^11^and pocket depths, were recorded. Only subjects with a PI of < 1 and residual pocket probing depth of ≥5 mm in all the teeth of at least two quadrants were finally included in the study. Four patients were excluded from the study after applying these criteria at the end of phase 1 therapy. Finally, 26 patients meeting these criteria underwent flap surgery of two different quadrants with an interval of 4 weeks. The quadrants were randomly assigned to the intervention (Diplen LX) and control (Coe-Pak^TM^) groups.

**Figure 1:**
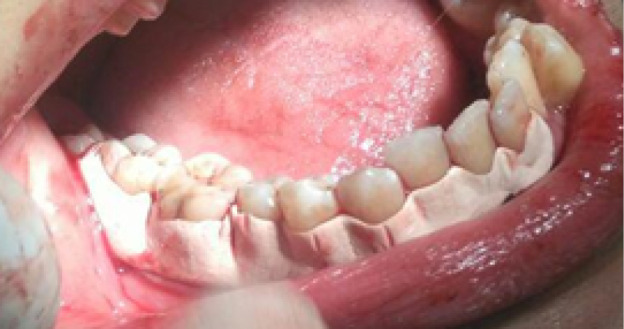
COE PAK Dressing used in control group.

**Figure 2:**
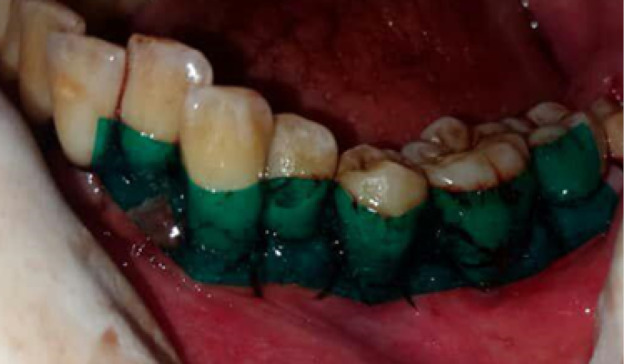
Diplen LX Dressing used in control group.

 Flap surgery was performed in each quadrant following the standard protocol of site preparation, incision, flap reflection, and thorough debridement. Minimal bone contouring was performed in some cases of both groups, while no case required any bone grafting. Primary closure was achieved using 3-0 silk suture on a 3/8 circle reverse cutting needle. Thereafter, in the control group, Coe-Pak^TM^ was placed at the surgical sites (Figure 1). Equal lengths of base and catalyst paste of this dressing were mixed on a glass slab according to manufacturers’ instructions. It was applied and pushed well into the embrasure spaces using moist gloved hands to mold it to the required contour. It was extended from one tooth mesial to the first suture to one tooth distal to the last suture of the surgical segment, extending from the cervical third of teeth to the mucogingival junction. Diplen LX dressing was placed in the intervention group (Figure 2). Occlusal clearance over the dressing was also checked. The extent of the dressing was the same as described above with Coe-Pak^TM^. In both cases, patients were given postoperative instructions and advised to rinse with 10 mL of 0.2% chlorhexidine gluconate solution twice daily for one week to control plaque. They were also prescribed ibuprofen tablets (600 mg three times daily for three days). On the 7th day after surgery, the periodontal dressing was removed in two parts (buccal and lingual) using a dental tweezer and a blunt probe. The patients were asked to fill an assessment questionnaire and rate the preferred dressing based on pain and discomfort experienced, taste, appearance, retention, burning sensation, and sensitivity experienced with each type of dressings. Wound healing index (WHI) parameter^12^was also evaluated at the surgical site on the 7th and 14th days after surgery in both groups.Patient-reported parametersincluded pain assessment based on the verbal rating scale and patient’s preference based on burning sensation, hypersensitivity, appearance, taste, and retention of dressings. This parameter was also evaluated on the 3rd and 7th days after surgery in both groups. Evaluation of wound healing was based on the parameters of tissue color, bleeding in response to palpation, the presence of granulation tissue, and the condition of incision margin. Each of these four parameters was separately assessed on the scale of 1 (very poor) to 5 (excellent), and the total score was finally divided by 4 to achieve the WHI score.

**Figure 3:**
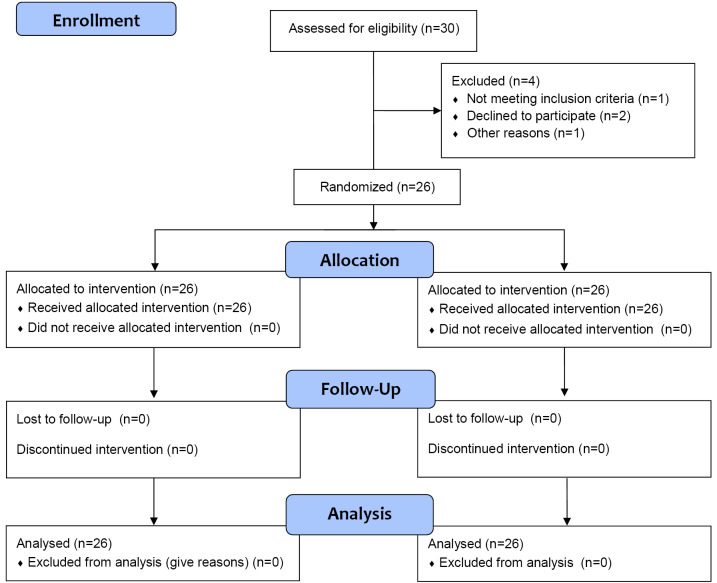
Consolidated Standards of Reporting Trials Diagram.

 To improve the reporting of RCT, we followed Consolidated Standards of Reporting Trials (Figure 3).

###  Data analysis 

 The study results were reported using descriptive statistical methods (mean ± standard deviation and frequency percentages). Chi-squared test was used to compare patients’ preferences in different degrees of pain, repair, and stages of periodontitis. Logistic regression was also used to predict patients’ preferences and control confounders. Statistical analysis was performed using SPSS 18, and the statistical significance was set at *P* < 0.05. Repeated measurements test was used to compare the severity of pain at different time intervals.

## Results

 The present study included 14 females and 12 males with a mean age of 33.15 ± 8.93 years. There were no statistically significant differences in clinical parameters (PI, mSBI, and PD) between the two groups at baseline. All the 26 patients reported on both the 3rd and 7th postoperative days after each surgery for pain parameter and on both the 7th and 14th days for WHI.

###  Pain assessment

Table 1 presents the evaluation of pain severity in the studied patients based on the VAS in the study groups on days 3 and 7 after surgery.

**Table 1:** Pain status in the studied patients

**Group**	**Days postoperatively**	**Mean±standard deviation**	**Min**	**Max**
Intervention group	3rd	1.12 ± 4.65	3	7
	7th	1.05 ± 1.19	0	3
Control group	3rd	1.51 ± 3.15	0	6
	7th	0.26 ± 0.45	0	1

 In the control group, the pain parameter on the 7th day after the surgery was significantly lower than the 3rd day by 3.46 units (*P* = 0.003).

 In the intervention group, the pain parameter on the 7th day after the intervention was significantly reduced by 2.88 units compared to the 3rd day (*P* = 0.001). In addition, the severity of pain in the studied patients was significantly lower in the intervention group than in the control group on the 3rd (*P* = 0.001) and 7th (*P* = 0.001) days after the intervention.

###  Wound Healing Index

Table 2 presents the evaluation of wound healing status in the studied patients based on the WHI in the study groups on days 7 and 14 after surgery.

**Table 2:** Wound Healing Index in the studied patients

**Group**	**Days postoperatively**	**Mean±standard deviation**	**Min**	**Max**
Intervention group	7th	0.59 ± 3.30	2.5	4.25
	14th	0.45 ± 3.84	3	4.5
Control group	7th	0.54 ± 4.05	3	4.75
	14th	0.24 ± 4.57	4.25	5

 In the control group, the WHI value on the 14th day increased significantly by 0.53 units compared to the 7th day after surgery (*P* = 0.001). In the intervention group, the WHI value increased significantly by 0.51 units on the 14th day compared to the 7th day after the intervention (*P* = 0.001). In addition, WHI values were significantly higher in the intervention group than in the control group on the 7th (*P* = 0.001) and 14th (*P* = 0.001) days after the surgery.

 In evaluating patients’ preferences in terms of the type of dressing, at the end of the study, of the 26 patients studied,‌three patients (11.5%) preferred Coe-Pak^TM^ dressing, and 23 patients (88.5%) preferred DiplenLX dressing. The patients reported their preferences in a questionnaire.

## Discussion

 Periodontal dressings have been introduced to reduce the risk of infection in the surgical area and bleed, improve the wound healing process, and reduce the pain and discomfort of patients. However, these features have been evaluated and criticized by several researchers in recent years. In addition, some clinicians believe in the use of periodontal dressings and their positive effect on patients, while some oppose the use of these dressings. In 2012, Genovesi et al^13^ reported that the use of periodontal packs effectively improved the results of non-surgical treatments in patients due to improved blood coagulation stability, no bleeding in the wound area, and reduced risk of bacterial infection in the surgical area.On the other hand, studies have shown that using these dressings is associated with increased plaque accumulation compared to cases in which no periodontal dressing is used.^14^ Therefore, the effect of different dressings on wound healing, plaque accumulation, and their biological compatibility with the tissue after surgery are the most important parameters affected by the type of material used in the dressing.^15^In Diplen LX, L indicates the word lidocaine, and X indicates dexamethasone. The analgesic property of this intervention is natural and logical due to the presence of lidocaine. In addition, the two studies examined and compared the effect of periodontal dressings and chlorhexidine gluconate on the healing of gingival wounds and surgical flaps, which significantly improved the results obtained with the use of periodontal dressings.^16,17^In the present study, patients with Coe-Pak dressing had a mean WHI of 3.84, and those with Diplen LX membrane dressing had a mean WHI of 4.57 on the 14th day after the surgery, which was significantly higher. Also, the pain scores in the patients in the Coe-Pak and Diplen LX groups on the 7th day after the surgery were 1.19 and 0.45, respectively, with much less pain in the Diplen LX membrane group. The results of the present study are consistent with a study by Ghanbari et al.^18^Periodontal packs cover the surface of the root and reduce pain after surgery. Contrary to the above findings, in studies by Moghare Abed et al,^19^Checchi et al,^20^and Bae et al,^21^similar pain was reported in patients with and without periodontal dressings after surgery, while in the study by Jones et al^17^ more pain was reported in patients following the use of periodontal dressings.However, the observed differences in the severity of pain can be due to the different severities of disease in the patients studied. On the other hand, bone density of patients at the site of surgery and the condition of patients’ gingiva is also factors involved. No facial edema was observed in the surgical area in the present study, and periodontal dressings had no effect in this category. In the study by Bae et al,^21^ unlike the present study, it was reported that edema of the face at the surgical site was also reported following the use of periodontal dressings.

## Conclusion

 According to the results of this study, despite the limitations of the study concerning the reporting of patients’ pain status subjectively, the severity of pain in both groups using periodontal dressings was low, with less severe pain in the Diplen LX membrane dressing group. In addition, the rate of wound healing was higher and excellent in the Diplen LX membrane dressing group according to WHI. Due to the above factors, the majority of patients preferred to use the Diplen LX membrane.

## Acknowledgments

 We would like to acknowledge the Department of Periodontics for their assistance.

## Authors’ Contribution

 HM initiated and conceptualized the research. HM, MS, and MF contributed to the design of the study. ZT, SHN, and TB performed the study and analyzed the data. All authors read and approved the final manuscript.

## Funding

 This study was supported by the Vice-Chancellor for Research, Faculty of Dentistry, Tabriz University of Medical Sciences.

## Ethics Approval

 This study was approved by the Research Ethics Committee of the Faculty of Dentistry, Tabriz University of Medical Sciences (IR.TBZMED.REC.1398.707).

## Competing Interests

 The authors declare no conflicts of interest.
